# The Role of Nutritional Factors and Intestinal Microbiota in Rheumatoid Arthritis Development

**DOI:** 10.3390/nu13010096

**Published:** 2020-12-30

**Authors:** Deshiré Alpízar-Rodríguez, Axel Finckh, Benoît Gilbert

**Affiliations:** 1 Research Unit, Colegio Mexicano de Reumatología, Mexico City 04318, Mexico; 2 Department of Rheumatology, Geneva University Hospitals, 1206 Geneva, Switzerland; axel.finckh@hcuge.ch (A.F.); benoit.gilbert@etu.unige.ch (B.G.)

**Keywords:** rheumatoid arthritis, risk factors, epidemiology, dietary intake, nutrition, microbiota

## Abstract

Evidence about the role of nutritional factors and microbiota in autoimmune diseases, and in rheumatoid arthritis (RA) in particular, has grown in recent years, however many controversies remain. The aim of this review is to summarize the role of nutrition and of the intestinal microbiota in the development of RA. We will focus on selected dietary patterns, individual foods and beverages that have been most consistently associated with RA or with the occurrence of systemic autoimmunity associated with RA. We will also review the evidence for a role of the intestinal microbiota in RA development. We propose that diet and digestive microbiota should be considered together in research, as they interact and may both be the target for future preventive interventions in RA.

## 1. Introduction

**Figure 1:**
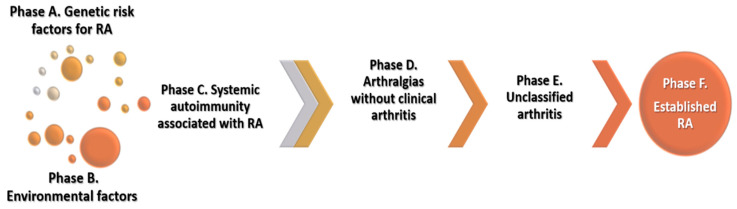
Proposed preclinical phases of RA development. Genetic, environmental factors and systemic autoimmunity interactions lead to RA development. The progression from one preclinical phase to another is not necessarily linear, and the phases may be overlapping.

Rheumatoid arthritis (RA) is the most prevalent systemic autoimmune inflammatory disease affecting approximately 1% of the adult population worldwide [1]. However, the etiopathogenesis of RA is only partially understood. The current knowledge is that in genetically susceptible individuals, environmental factors induce a pathological activation of the immune system that eventually leads to clinical onset of RA [2]. The European League Against Rheumatism (EULAR) has proposed a terminology for specific preclinical phases of RA development, which are not necessarily consecutive or mutually exclusive (Figure 1) [2,3]. Interactions between genetic factors, environmental factors and the presence of autoantibodies lead to increased risk of developing RA.

Several studies suggest environmental factors play an important role in the etiology of the disease [4,5]. Smoking is the environmental factor more consistently associated with RA development [6]. However, patients are frequently also concerned about the effect of diet on the development of RA. Human diet has gone through extensive transformation globally, with increasing consumption of processed foods, salt and carbohydrate enriched products, contributing to the development of obesity and other chronic diseases, such as hypertension, type 2 diabetes mellitus and cardiovascular diseases. Some nutritional factors may contribute to the pathologic activation of immune system, eventually leading to RA, and some others may be protective. Studies have suggested that the initial steps of the pathological autoimmune response associated with RA take place at mucosal sites, such as intestinal or airway mucosa, rather than in the joints [7], and are associated with higher abundance of particular bacterial species [8]. Diet modifications affect the composition and function of the intestinal microbiota and it is possible that part of the observed effect of nutrition on RA is mediated by changes in the microbiota.

Although a number of studies have analyzed the impact of dietary factors and intestinal dysbiosis in RA development, many controversies remain. As many studies are performed cross-sectionally, it is often impossible to establish whether the described associations are causal or not. The aim of this manuscript is to review the role of nutritional factors and of intestinal microbiota in RA development. We will focus on selected dietary patterns, individual foods and beverages that have been most consistently associated with preclinical phases of RA, such as ‘systemic autoimmunity associated with RA’ and with the disease itself, whether protective or conferring an increased risk. We will then review the evidence of a role of the microbiota in RA development. We will discuss these associations in pre-clinical phases and in established RA separately. Associations reported in established disease are prone to reversed causation, which occurs when the disease status influences exposure, and could bias the association of dietary factors or microbiota observed in established RA [9]. The relevance of studying the role of diet and intestinal microbiota in RA development lies in their potential to be modified and to be used in preventive strategies.

## 2. Nutrition and Development of Systemic Autoimmunity Associated with RA

‘Systemic autoimmunity associated with RA’ is a pre-clinical phase of RA, often considered the immune onset of the disease and characterized by the presence of autoantibodies, such as the rheumatoid factor (RF) and anti-citrullinated protein antibodies (ACPAs). Few studies have analyzed the impact of nutritional factors on the development of systemic autoimmunity associated with RA, in individuals at risk of RA.

### Omega-3 and Omega-6 Fatty Acids

Omega-3 fatty acids have been suggested to be protective against the development of autoimmunity associated with RA. In a nested case-control study in the Studies of the Etiology of RA (SERA), healthy FDR-RA individuals who developed ACPAs had used less frequently omega-3 supplements (Odds ratio, OR 0.14, 95% Confidence Interval, CI 0.03–0.68) and had significantly lower concentrations of omega-3 fatty acids in red blood cell membranes than controls (30 cases vs 47 controls) [10]. The SERA research group further analyzed, in a larger number of FDR-RA individuals, whether omega-3 fatty acids were also associated with RF and whether these associations were modified by shared epitope (SE) positivity. Individuals with RF and SE positivity or with ACPA and SE positivity had lower concentrations of omega-3 fatty acids in red blood cell membranes (OR 0.27, 95% CI 0.10–0.79 and OR 0.42, 95% CI 0.20–0.98, respectively) [11]. These results suggest a potential protective effect of omega-3 fatty acids on RA-related autoimmunity, which may be more prominent in those with genetic susceptibility to RA.

## 3. Nutrition and Development of RA

Several studies have analyzed associations of dietary patterns, individual foods and beverages with established RA. We are going to review potentially protective and hazardous factors and discuss controversial factors.

### 3.1. Protective Factors

#### Alcohol

In animal models, adding small doses of ethanol to mice’s drinking water delays the onset of collagen-induced arthritis, suggesting preventive properties of low dose and persistent alcohol consumption [12]. In humans, moderate alcohol consumption (defined as 5.0–9.9 g/day) has been described as a protective factor against RA [13,14]. A meta-analysis of nine observational studies found a protective effect of alcohol on the development of RA (OR 0.78, 95% CI 0.63–0.96), and even more pronounced in ACPA-positive RA (OR 0.52, 95% CI 0.36–0.76) [15].

### 3.2. Hazardous Factors

#### 3.2.1. Salt Consumption

High salt consumption has been suggested a risk factor for the development of RA, in particular in smokers [16,17]. In a nested case-control study from Sweden, 386 patients with RA were compared to 1886 matched controls [18]. High sodium intake doubled the risk of RA among smokers (OR 2.26, 95% CI 1.06–4.81) but not in nonsmokers. A study by same authors compared ACPA positive RA vs ACPA negative RA, and after stratification by salt consumption, ever-smokers with medium to high sodium consumption had an increased risk of ACPA-positive RA (OR 1.7, 95% CI 1.2–2.4) [19]. In a Spanish cohort study of 18,555 individuals, 392 persons developed RA [20]. Persons with high daily sodium intake (>4.55 g) had a higher risk of developing RA adjusting by confounders, such as physical activity, hypertension, cardiovascular diseases, diabetes, cancer and smoking (OR 1.5, 95% CI 1.1–2.1). However, in this study nonsmokers had a higher association than ever smokers.

#### 3.2.2. Sugar-Sweetened Beverages

In the Nurses’ Health Study (NHS), regular consumption of sugar-sweetened sodas, meaning >1 daily serving, significantly increased the risk of developing RA [21]. The association was independent of obesity and other socio-economic factors and tended to be stronger for late-onset RA (HR 2.64, 95% CI 1.56–4.46). No causal relation was found with diet soda or between sugar-sweetened soda and seronegative RA [21]. An interaction between sugar sweetened soda consumption and smoking was described.

### 3.3. Controversial Factors

Despite the large number of studies examining the role of individual foods, dietary factors, dietary supplements and beverages in the development of RA, many controversies remain.

#### 3.3.1. Controversial Dietary Factors

- Mediterranean diet is characterized by high consumption of vegetables, legumes, olive oil, alcohol, and fish. This dietary pattern has been associated with a number of chronic diseases, including RA. In the NHS, 913 incident cases of RA were documented during 3,511,050 cumulative person-years of follow-up. After adjustment for several lifestyle and dietary variables, adherence to Mediterranean dietary pattern was not associated with increased risk of RA in women [22]. A nested case-control study in the Swedish EIRA cohort, analyzed data of 1721 patients with incident RA and 3667 controls and found that a Mediterranean diet was inversely associated with the risk of RA, particularly among men (OR 0.49, 95% 0.33–0.73) and with RF and ACPA positivity (OR 0.69, 95% CI 0.54–0.88 and 0.72, 95% IC 0.57–0.92, respectively) [23]. Recently, a French cohort, the E3N study identified 480 incident cases among 62,629 women and found that a Mediterranean diet was associated with a decreased risk of RA among ever smokers (HR 0.86, 95% CI 0.84–0.99) [24].
- Meat and dairy products consumption. During 12 years of follow-up in a Swedish cohort study (381,456 person-years), 368 individuals developed RA. No associations between the development of RA and the consumption of meat and meat products or total consumption of milk and dairy products were found (HR 1.08, 95% CI 0.77–1.53 and HR 1.09, 95% CI 0.76–1.55, respectively) [25]. Other analyses related to meat consumption are ongoing, such as a large prospective Danish cohort which aims to investigate the impact of fiber, red meat and processed meat on risk of late-onset chronic inflammatory diseases, including RA [26].
- Vegan diet. Vegan diet has been associated with reduced inflammation markers. In a randomized control trial, markers of inflammation relevant for RA were compared in individuals who were on vegan diet against individuals on meat-rich diet during four weeks. Vegan diet reduced neutrophils, monocytes and platelets related to branched-chain amino acids. These findings suggested a mode of action via the mTOR signaling pathway [27]. Another study reported improved signs and symptoms of RA with a gluten-free vegan diet and the effects on arthritis correlated with a reduction in antibodies to food antigens [28]. However, no protective effect of vegan diet on RA development has been demonstrated.
- Fasting has been reported as beneficial on RA disease activity [29,30,31]. A systematic review reported 31 studies examining the effects of fasting in patients with RA, but only four controlled studies analyzed follow-up data over at least three months after fasting, and showed statistically and clinically significant beneficial effects [32]. However, a protective effect of fasting on RA development has not yet been demonstrated.
- Elemental diet. A small study compared elemental diet with oral prednisolone for 2 weeks in RA patients. Elemental diet appeared as effective as a course of oral prednisolone 15 mg daily in improving subjective clinical parameters of RA [33]. In a smaller but longer study, patients with active RA were randomized either to a liquid elemental peptide-diet for four weeks or usual diet. Elemental diet produced transient but statistically significant improvement in pain and disability measured with Health Assessment Questionnaire (HAQ)-score [33]. Similarly, to the vegan diet and fasting, the role of elemental diet on RA development has not been explored.

#### 3.3.2. Omega-3, Omega-6 Fatty Acids and Fish Consumption

A study compared polyunsaturated fatty acids (PUFA), including omega-3 and omega-6, between pre-RA individuals (measurement prior to disease onset) and matched controls from the European Prospective Investigation into Cancer and Nutrition (EPIC). Omega-6 PUFA levels of the erythrocyte were inversely associated with risk of RA, but no association were observed for omega-3 [34]. However, in a large cohort study from Sweden, in which 205 RA cases among 32,232 women were recorded, a similar analysis supported the hypothesis that omega-3 PUFA may play a role in RA development. In this study, long-lasting intake of omega-3 fatty acids higher than 0.21 g/day decreased the development of subsequent RA by 52% (95% CI 29–67%) [35], as well as a regular consumption of fish at least once per week (risk ratio (RR), 0.71, 95% CI 0.48–1.04). A meta-analysis examining the association between fish consumption and subsequent development of RA suggested a trend towards a protective effect with one to three portions of fish per week (RR 0.76, 95% CI 0.57–1.02) [36]. In a large prospective cohort study with 1080 incident RA cases in 3,863,909 person years of follow-up, no clear protective effect of omega-3 fatty acids intake on RA risk was found. However, authors reported a significant interaction between tobacco smoking and fish consumption. Frequent fish consumption among ever smokers women attenuated the strong association of smoking and RA, particularly in young-onset RA (diagnosed at 55 years of age or younger) [37].

#### 3.3.3. Vitamin D

Vitamin D has immunomodulatory properties [38]. Low vitamin D levels may contribute to increased immune activation and may lead to RA development [39]. Several studies have reported vitamin D deficiency in RA patients, in up to 76% of patients and inverse association between vitamin D levels and disease activity [39,40,41]. However, the evidence is controversial as reverse causation may explain some of these findings and a beneficial effect of vitamin D supplementation on RA disease onset has not been demonstrated.

#### 3.3.4. Coffee and Tea

In the large prospective NHS, the authors did not find a significant association between coffee, decaffeinated coffee, or tea consumption and the risk of RA in women [42]. Another prospective cohort study reported decaffeinated coffee consumption (≥4 cups by day) was associated with increased RA onset (RR 2.58, 95% CI 1.63–4.06), while tea consumption was inversely associated with RA (RR 0.39, 95% CI 0.16–0.97) [43].

#### 3.3.5. Obesity

The role of obesity as a risk factor for RA is controversial, as it has been described as a risk factor in women, but as a protective factor in men [13,44,45]. Obese women (BMI ≥ 30.0 kg/m^2^) in the NHS tended to have an increased risk of RA, particularly those diagnosed at younger ages (HR 1.65, 95% CI 1.34–2.05) and in those obese during adolescence (HR 1.35, 95% CI 1.10–1.66) [13]. Similar results were found in Europe, with obesity increasing the risk for seronegative RA in women (HR 1.6, 95% CI 1.2–2.2) [44]. In men, the effect of obesity was less obvious and some studies have even described a reduced risk of RA in men [45]. From two large population-based health surveys (30,447 and 33,346 participants), excess weight or obesity in men was associated with a reduced risk of RA development (OR 0.33, 95%CI 0.14–0.76 and OR 0.60, 95%CI 0.39–0.91, respectively) [45].

## 4. Gut Microbiota in RA Development: Is Microbiota the Missing Link between Nutritional Factors and RA Onset?

### 4.1. Human Gut Microbiota in RA

Even though triggering of RA by micro-organisms has been speculated as early as 1896 [46], researchers only recently renewed interest in this hypothesis. Modern utilization of 16S and “shotgun” sequencing allowed comparison of healthy-control gut flora with microbiota from RA patients (Table 1). The subsequent findings have not been completely consistent across the literature, but a repeated feature has been the increase of *Prevotella* species in early RA [47,48,49,50], associated to a relative decrease in the Bacteroides genus [48,51]. The overrepresentation of *Prevotella* species is usually no longer found in established and treated RA patients [52,53,54]. In particular, *Prevotella copri* raised researcher’s interest, because of worsening arthritis when transferred in mice models [47,48]. To better distinguish causality from fortuitous association, Alpizar-Rodriguez et al. have studied gut microbiota in first-degree relatives of RA patients, who did not have the disease. They demonstrated an enrichment of *Prevotella copri* among individuals who displayed auto-immunity associated with RA or specific articular symptoms, compared to healthy seronegative subjects [8]. P. Wells et al. have further confirmed that healthy individuals genetically at risk for RA tended to host increased proportions of *Prevotella* species [55]. However, the specific expansion of *P. copri* has only been observed in pre-clinical stages or early untreated RA [55].

Some researchers have proposed a gut flora classification into “enterotypes” [56]. Even if this concept is still largely debated, an enterotype dominated by *Prevotella* species is often mentioned [57]. Given published association between RA genetic risk and *Prevotella* species, [55] we hypothesize that subjects genetically at risk for RA may simply be more susceptible to host gut flora enriched with *Prevotella* species. Subsequently, this “*Prevotella*-driven” microbiota would shift to a “pro-inflammatory” conformation, characterized by higher prevalence of *P. copri*, driving the progression to RA. Beyond mice models, the hypothesis of a pro-inflammatory role of *P. copri* is further supported by immune reactivity against *P. copri* peptides in RA subjects [58,59]. Nevertheless, additional immunological studies on human samples (blood, stool, or gut biopsies) are necessary to clarify the mechanism linking *P. copri* to the onset of RA. Finally, other bacterial species have been associated with human RA, such as *Collinsella aerofaciens* [53,54]. The latter also worsens arthritis in mice models [53,54], which underlines the fact that there may not be a single bacterial culprit, but probably a complex interplay between several microorganisms and host defenses.

### 4.2. Hypothesis Linking Gut Microbiota to RA

The intestinal epithelium layer and the underlying structures of the intestinal barrier protect the organism against invasion by micro-organisms and their toxins, while allowing the absorption of important quantities of nutrients and fluids. This selective permeability is modulated by various mechanisms, most of which remain to be understood [60].

Biological markers of the intestinal barrier function, such as zonulin, have been identified [61]. They allowed linking impaired intestinal barrier function to diseases, such as celiac disease [62] or depression [63]. Evidence for increased gut permeability in established RA is weak [30,64,65], and mostly biased because of non-steroidal anti-inflammatory drugs (NSAIDs) use. Indeed, NSAIDs are known to induce small bowel lesions, referred to as NSAID-induced enteropathy. These small bowel lesions include mucosal breaks [66], which are surprisingly prevalent in RA patients who chronically take NSAIDs [67]. Recent studies have demonstrated increased gut permeability both in early and in established RA patients, using zonulin as a serological marker [68]. This increase in gut-permeability has been demonstrated in about 1/3 pre-RA patients [68]. This finding was further supported by ileus mucosal biopsies of RA patients, manifesting lower expression of tight-junction proteins and increasing levels of immune cells in the lamina propria compared to healthy controls [68]. To evaluate potential bacterial translocation, Ayyappan et al. [69] compared serum from RA patients to healthy age-sex matched controls and assessed various serological antimicrobial response factors. Among others, these authors found significant elevation of sCD14, as previously reported [70,71], higher levels of LBP and Lysozyme [72], in line with the hypothesis of an increased intestinal permeability in these patients.

Beyond *P. copri* or *C. aerofaciens* isolates, the “whole” pre-RA or RA-derived microbiota also worsen arthritis when transferred to mice models [47,51,53]. For instance, *Prevotella* species have been found invading the mucus layer in irritable bowel disease models [73] or in human colonic cancer related studies [74,75]. As suggested by Palm et al. [76], the ability of particular strains to invade a normally sterile environment could be the common denominator of “auto-immunogenic” or “pro-inflammatory” microbes. Conversely, some species of *Prevotella* exert protective effects, attenuating arthritis in mice [77]. This illustrates that besides *P. copri*, other bacteria are capable of modulating intestinal epithelium integrity and have beneficial immunomodulatory effects.

More recently, Balakrishnan et al. [78] demonstrated on a humanized mouse model that gavage with some RA-associated bacteria (*Eggerthella lenta* or *Collinsella aerofaciens*) also increased the gut permeability, compared to gavage with non-associated bacterial species (*Prevotella histicola* or *Bifidobacterium* sp.). Their results confirmed that RA-associated bacteria are not just “inflammation-associated” taxa, but rather active contributors to the chronic inflammatory state in mice. Tajik et al. studied a mice model of collagen-induced-arthritis [68] and demonstrated that intestinal inflammation and increased gut permeability precede the onset of arthritis. Interestingly, mice-to-mice fecal microbiota transplantation also transferred the leaky barrier and mucosal inflammation [68]. Finally, they were able to attenuate the development of arthritis by preventively targeting intestinal barrier dysfunction, using butyrate or zonulin-inhibitors [68]. If future research confirms gut permeability as a relevant therapeutic target in RA, various interventions could be considered to modulate the epithelial gut barrier function (Table 2).

The role of hormonal factors in the female over-representation of RA is not fully understood [79,80]. Beyond estrogen bioavailability, the increased female risk of RA could also be linked to gut microbiota. While to date, little is known about the contribution of sex-dependent differences in human microbiota in RA, sex-specific variations in gut microbiota are well established [81], and some intestinal bacteria can interact with sex-hormones [82]. Mice models suggested a contribution of the microbiota to the female sex-bias of auto-immune diseases, since female mice with arthritis had significantly less microbial diversity than male individuals and a different microbiota composition [83]. Moreover, non-obese diabetic female mice usually have 1.3 to 4.4 times higher incidence of type 1 diabetes (T1D), but germ-free mice loose this gender bias (female to male ratio 1.1–1.2) [82]. Transfer of gut microbiota, in mice models, from adult males to immature females, produced an elevation of testosterone and metabolomic changes resulting in a robust T1D protection [84].

### 4.3. The Gut-Joint Axis and Autoimmunity Onset

Hypotheses linking “pro-inflammatory” microbes to RA generally suppose an activation of autoreactive T-cells in the intestine, which later migrate to the joints and exacerbate inflammation. Such systemic spread of local immune cells have been shown by Teng et al. in the K/BxN mice model [103]. Indeed, arthritis triggered by segmented-filamentous-bacteria-containing feces was actually driven by the migration of T-follicular-helper cells from the Peyer-Patches to the peripheral lymphoid tissues; where auto-antibodies are produced [103].

Strong evidence for immune cell migration from the gut to the joints is still lacking in human RA. However, patient-derived gut immunoblasts have been known for a long time to strongly bind high-endothelial-venules of synovium [104,105]. This could partly explain the tendency for gut-joint axis in rheumatic disease. Interestingly, May et al. have shown in one enterogenic-SpA patient that a few expanded T-cell clones in the inflamed synovium were also overrepresented in the gut and in the peripheral blood [106]. The latter suggests that the systemic spread of mucosal expanded T-cell clones occurs in humans; but similar findings have yet to be established in RA.

Once the gut permeability has been impaired, several mechanisms are suspected to activate self-reactive T-cells, linking auto-immunity with microbial homeostasis:

- Molecular mimicry: A loss of tolerance against autoantigens because of structural similitude with a bacterial antigen. A classic example is rheumatic fever, which follows a group A Streptococcal infection. Several structural similarities with human proteins characterize Group A Streptococcus M5 protein; for instance, cardiac myosin, which will then also be targeted by antibodies generated against the Streptococcus [107,108]. A similar cross-reactional mechanism is also suspected to occur with intestinal bacteria in less acute contexts, such as lupus [109] or anti-phospholipid antibody syndrome [110]. After the auto-immune response is initiated, it can amplify itself by subsequently targeting other adjacent epitopes (“epitope spreading”).
- Neo-autoantigens generation: This represents a variant of the cross-reaction described above. The "cross-reactive" epitope, instead of being a bacterial structure, would be a human epitope modified by a microbe or by an inflammatory reaction of the host against the microbe (for instance: A citrullinated protein) [111].
- Activation of dual TCR cells: Some T-lymphocytes are known to have two T-receptors, with two different affinities. One of these receptors could have an affinity for a ’self’ structure’, but the T-cell would be activated via its other T-receptor, recognizing a bacterial epitope. The subsequent immune reaction against the microbial peptide could then collaterally target host structures [112,113].
- A-specific activation: Some bacterial antigens can a-specifically activate lymphocytes in a via innate immunity receptors (e.g., the "Toll Like Receptors"). If these lymphocytes have T receptors with affinity for host structures, their "accidental" activation by this non-specific mechanism could trigger autoimmune disorders [111,113].
- Antigen dissemination: A damaged intestinal mucosa allows bacterial components or even whole microorganisms to be translocated into the circulation («leaky gut» [114]). It is not uncommon to find these immunogenic structures in serum or synovial fluid [115]. This may contribute to local inflammation (i.e., if antigen has a tropism for synovium) or loss of tolerance [116].

### 4.4. Interaction between Diet and Microbiota in RA

Diet is a key factor able to shape gut microbiota. However, most of the interactions between specific diets and resulting gut-microbiota alterations remain poorly understood and published findings can be conflicting. The existing experiments aimed at shaping the gut microbiota using diet have focused mainly on the metabolic syndrome. Some findings might, however, be interesting for the field of RA. For instance, healthy subjects exhibit improved glucose metabolism after a diet supplemented with barley kernel-based bread (very rich in fibers) and display an expanded prevalence of *Prevotella* species, specially *P copri* [117]. This association of *P. copri* with a beneficial effect contradicts the observed ability of *P copri* to drive insulin resistance in type II diabetic patients and in mice models [118]. We also report above that Mediterranean diet seems to be protective for RA, while this diet is also known to increase the abundance of fiber degrading bacteria, such as specific *Prevotella* species [119]. A strain-level analysis could help make sense of this contradictory findings. Indeed, diets rich in fibers may select *P. copri* strains with higher potential for complex carbohydrate degradation, while *P. copri* strains associated with an omnivore diet showed a higher prevalence of genes related to branched-chain-amino-acid (BCAA) biosynthesis, and subjects harboring these genes in their microbiome had a higher BCAA urinary level [120] (circulating BCAA are associated with metabolic syndrome [121]). The latter underlines the necessity of a strain-level-identification approach to study association between *P. copri* and RA. Indeed, using shotgun sequencing, Scher et al. revealed that new onset RA patients do not exactly host the same strains as control patients [48].

The beneficial effect of dietary fibers is probably the best studied interaction linking diet with microbiota composition [122]. Beyond selective growth of beneficial strains, the production of short-chain-fatty-acids (SCFA) by the gut flora from the ingested plant fibers is also a key element. SCFA are the main energy source of colonocytes [123] and are currently studied for various other beneficial effects, such as regulating IgA secretion [124] or improving intestinal barrier function [68,100]. Based on these findings, Häger et al. conducted the first feasibility study of fiber supplementation in RA patients [102]. The preliminary results showed reduced serum zonulin and calprotectin levels at the end of the follow-up, as well as modest improvement in physical function and quality of life (*n* = 36 patients) [102]. The beneficial effect of fiber supplementation remains to be confirmed in larger studies and the long-term benefit of diets on the microbiota better understood. For instance, a whole-grain diet in obese individuals led to reduced body weight and inflammatory markers, but authors did not observe major changes in microbiota composition after eight weeks [125]. Similarly, Fragiadakis noticed significant changes in gut microbiota after low-carb or low-fat diets, but these changes tended to regress to baseline after one year of follow-up, despite maintenance of diet by participants [126]. This underlines that the gut microbiota might be more resilient to dietary changes; or that significant changes occur on the functional level, which are not identified by 16S-based methodology.

**Figure 2:**
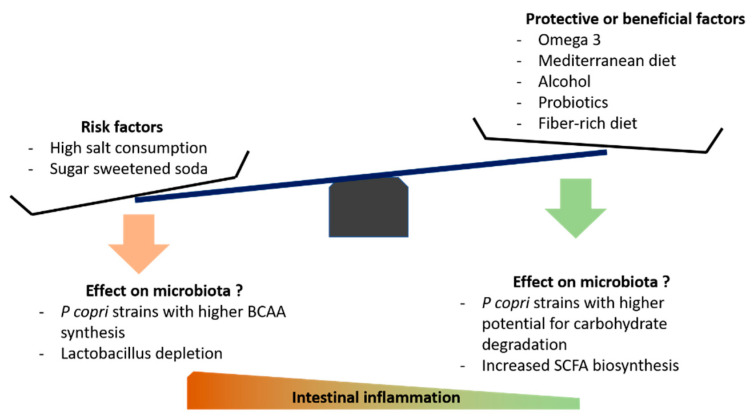
Some putative dietary factors involved in the risk of RA development and their potential effects on the microbiota. “Beneficial factors” refers to interventions that have shown modest but positive effects in established RA. BCAA—Branched Chain Amino Acids. SCFA—Short Chain Fatty Acids. *P copri*—*Prevotella copri.*

Beyond diets, specific nutrients may also impact the composition of the gut microbiota. Mice model with serum-transfer induced arthritis have increased intestinal permeability, plasma endotoxins, and a reduced intestinal concentration of omega3-derived cytokine called “resolvin” [99]. Subsequent inoculation with *Porphyromonas gingivalis* exacerbated these changes, while administration of the resolvin restored the barrier function and was associated with reduced joint inflammation and swelling [99]. This could elegantly explain the observed beneficial effect of omega-3 supplementation [127]. Salt consumption has also been linked with microbiota alterations, in both murine models and human subjects [128,129]. Notably, Wilck et al. have demonstrated that a high-salt diet in mice markedly shifts gut microbiota, in particular reducing the abundance of *Lactobacillus* species [129], and associates with an increase in splenic Th17-cells. A 14-day high-salt diet challenge in healthy male volunteers reproduced this *Lactobacillus* depletion and elevated circulating Th17 cells [125]. Salt consumption and relation with microbiota in the context of RA have never been studied, but it might be a relevant question, since Th17 cell involvement is a recurrent finding in mice models of arthritis [47,130,131,132]. How moderate alcohol consumption could be a protective factor for RA is not fully understood. However, Caslin et al. have recently studied the effect of moderate alcohol intake in a mice model of autoimmune encephalomyelitis [133]. Surprisingly, moderate alcohol intake induced greater disease remission in male mice; the latter seems to associate with sex-specific gut microbiota alteration [133]. Alcohol consumption has also been identified as a protective factor in human RA [134]. The benefit of ethanol in arthritis mice models is well-established, and was recently shown to result from a direct effect on T follicular helper cells [135]. A possible link to the gut microbiota has not been studied. Finally, probiotics such as *Lactobacillus* species are known to exert beneficial effects in arthritis mice models [136,137,138,139]. However, effectiveness in treating established RA is still unclear. Mohammed et al. have conducted a meta-analysis including 361 patients from 6 randomized trials (probiotics tested—*Lactobacillus* species, *Bifidobacterium bifidum* and *Bacillus coagulans*) [140]. They concluded that probiotic supplementation reduced reported levels of IL-6, but did not significantly change disease activity [140]. To our knowledge, no human trial has tested probiotics in a preventive or pre-RA context, nor assessed the effect of other potentially beneficial species such as *Prevotella histicola* [77]. Figure 2 shows putative dietary factors involved in RA development and their potential effects on the microbiota.

## 5. Discussion and Future Perspectives

A number of clinical studies have examined the role of diet in RA development. However, due to study design and the struggle to differentiate diet from associated confounding factors (i.e., healthy lifestyle or socio-economic status), it is often problematic to establish whether the associations between diet and RA are causal or not. The rare interventional studies focusing on diet in RA are hampered by low sample size, poor methodology, short-term follow-up, and a focus on established RA populations. Other explanations for the contrasting results of the literature relating to diet may be the diverse dietary interventions, and the difficulty to measure the real consumption of individual foods. In clinical studies, a single type of food or nutrient may confer only a modest effect, difficult to demonstrate reliably, unless several dietary factors are grouped together and demonstrate a stronger combined effect [141,142]. To allow appropriate causal inferences, future research needs to overcome imperfect diet-compliance and limited follow-up of nutrition clinical trials. One possibility could be to improve existing “artificial guts” to study the impact of dietary changes. Edward G. et al. were able to study microbiota changes following a fat-only diet in such a setting [143]; experiments that could help understand how dietary patterns, or even fasting, could selectively promote or reduce the abundance of a given bacterial taxa. Finally, intestinal inflammation associated with specific ‘dysbiosis’ suggests new possible targets. A recent study suggested that supplementation with intestinal alkaline phosphatase, which in animal models can reduce lipo-polysaccharide production, may decrease intestinal inflammation [144]. There is only limited evidence about the effectiveness of such intervention in humans, but the administration of exogenous alkaline phosphatase to patients with ulcerative colitis was well tolerated [145].

It is simplistic and reductive to systematically refer to the “microbiota” to explain the unknown. However, given its role at the crossroads of many metabolic systems, the gut microbiota in relation to intestinal epithelial homeostasis is a promising key element to articulate various risk-factors interactions. As rightfully underlined by Harald Brüssow [146], the whole “microbiome” research field has partly become entangled in terminological and logical issues. In particular, the term ”dysbiosis” is pointed out as spurious or misleading, since it lacks proper consensual definition. This vague concept of “an imbalanced” gut flora leads to probably spurious conclusions, since case-control studies implicitly label any microbiota differences observed in diseased individuals, compared to healthy controls, as “dysbiosis”. We should rather postulate that a variety of bacterial species could have the ability to promote auto-immunity, as they interact with gut mucosa and local lymphoid organs. *P. copri* and *Collinsella aerofaciens* gained credibility as potential “pathobionts”, since worsening arthritis when transferred to mice models. Adequate mice studies should, for each identified bacterial taxa, confirm the “arthritogenic” potential. Ideally, these pro-inflammatory pathobionts should also be studied on human gut mucosa samples. Selective fluorescent marking could help confirm the extent to which they invade the mucus layer or submucosa.

We have listed several individual foods, dietary patterns, dietary supplements, and beverages that have been associated with RA development in clinical studies. An appealing hypothesis would be that these dietary factors are effective by modifying the gut microbiota and modulating the intestinal barrier integrity, thus changing the antigenic load and subsequent immune dysregulation. While the evidence suggesting a role of nutritional factors in RA disease progression and outcomes is increasing, randomized trials with “anti-inflammatory” diets in established RA have only shown modest effects [147,148,149]. Bustamante et al. are attempting an approach that would optimize the nutritional strategy based on evidence [150]. Dietary counseling in conjunction with disease modifying anti-rheumatic drugs could become part of the management of established RA. Other targeted preventive strategies could also be proposed to manipulate microbiota, such as male to female FMT, or supplementation with a combination of nutrients and prebiotics, to obtain a synergistic effect. Before a “preventive” diet for RA can gain acceptance, clinical trials during the pre-clinical stages of RA are needed to establish if lifestyle-related interventions are ultimately able to prevent or delay the onset of RA in high risk individuals.

## 6. Conclusions

Although published literature is still limited, interest in the role of diet and microbiota in the development of RA is growing. Several studies have recently suggested that the use of omega-3 and moderate alcohol consumption may have a protective effect on RA development, particularly among smokers or individuals at high risk. We postulate that the microbiota and intestinal barrier homeostasis may be a missing link between the various nutritional factors and the development of RA. Modification of microbiota using dietary interventions and focusing on the improvement of the intestinal barrier function may become an important part of the future “preventive” nutritional strategies. Longitudinal cohort studies during the preclinical phases of RA, studying dietary patterns and microbiota changes concurrently, are needed to better understand the causality of these associations. Clinical trials in individuals at risk of RA need to be conducted in order to determine the feasibility and the efficacy of such interventions.

## Figures and Tables

**Table 1:** Up-to-date Human analysis of gut microbiota in rheumatoid arthritis (RA).

Author/Year/Ref.	Cases	Controls	Method	Results
Scher et al., 2013 [48]	New onset RA patients (*n* = 44)	Chronic RA patients (*n* = 26); Healthy controls (*n* = 28); Psoriatic arthritis (*n* = 16)	16S sequencing; Shotgun sequencing on a subset of 44 samples; Antibiotic treated mice model.	Specific *P. copri* increase in NORA. *P. copri* mice colonization increased sensitivity to chemically induced colitis.
Zhang et al., 2015 [52]	RA patients: Naïve Treatment (*n* = 94); DMARD Treatment (*n* = 21)	Healthy controls (*n* = 97)	Shotgun sequencing (fecal, dental, and salivary samples).	Concordance between gut and oral microbiomes. *Haemophilus* spp. depleted in RA, at all three sites, and negatively correlated with serum auto-antibodies. *Lactobacillus salivarius* over-represented in RA. No significant difference in *P. copri*.
Maeda et al., 2016 [47]	RA patients (*n* = 17)	Healthy controls (*n* = 14)	16S sequencing; SKG germ-free mice model	1/3 of RA microbiota dominated by *Prevotella* species (80% *P. copri*). “*Prevotella*-dominated” RA microbiota increased arthritis severity in mice.
Chen et al., 2016 [53]	Treated RA patients (*n* = 40)	Asymptomatic FDRs (*n* = 15); Healthy controls (*n* = 17)	16S sequencing; Human epithelial cell line and humanized mouse model of CIA	Decreased gut microbial diversity in RA. *Eggerthella* and *Collinsella* genus associated with RA; decrease of *Faecalibacterium*. No association between RA and *Prevotella copri*. *Collinsella aerofaciens* increased incidence and severity of mice arthritis, as well as intestinal permeability.
Alpizar-Rodriguez et al., 2019 [8]	Seropositive or symptomatic FDRs (*n* = 83)	Asymptomatic FDRs (*n* = 50)	16S sequencing.	Enrichment *Prevotella copri* in the ’pre-RA’ group. Decreased *Oxalobacteraceae*. No difference in alpha or beta diversity.
Jeong et al., 2019 [49]	Pre-clinical and untreated NORA (*n* = 29)	Healthy controls (*n* = 25)	16S sequencing.	*Prevotella* genus slightly enriched in RA and pre-RA subjects. *Collinsella* decreased in RA.
Kishikawa et al., 2020 [50]	RA (71% untreated)(*n* = 85)	Healthy controls (*n* = 42)	Shotgun sequencing.	Enrichment of *Prevotella* species in the RA. No difference in overall diversity.
Mena-Vazquez et al., 2020 [54]	Stable RA patients (*n* = 40)	Healthy controls (*n* = 40)	16S sequencing.	No difference in diversity. *Enterococcus*, *Sedimentibacter*, and *Collinsella* significantly more frequent in RA. Decrease in *Sarcina* and *Porphyromonas*. *Collinsella aerofaciens* increased in RA patients.
Y. Tong et al., 2020 (abstract) [51]	High-risk pre-Ra ACPA positive (*n* = 42); RA patients (*n* = 31)	Healthy controls (*n* = 38)	16S sequencing. CIA mice model.	*Bacteroidaceae* abundance decreases in pre-RA and RA. Mice CIA more severe after FMT with stool from pre-RA. Associates with reduced gut barrier function and intestinal epithelial damage.

RA—Rheumatoid Arthritis. NORA—New-Onset Rheumatoid arthritis. DMARD—Disease Modifying Anti-Rheumatic Drugs. FDRs: First-Degree Relatives of RA patients. CIA—Collagen-Induced Arthritis. FMT—Fecal Microbiota Transplantation.

**Table 2:** Main factors influencing intestinal barrier function.

Impairing the Intestinal Barrier (Increased Permeability)
NSAID (human data) [65].
Hyperglycemia (mice data) [85]
Gliadin (mice data), increases intestinal permeability and exacerbates NSAID-induced small-intestinal damage. (but did not increase the mRNA expression levels of IL-1β and TNF-α and did not induce visible small-intestinal damage when given alone) [86].
Acute stress (mice data) [87].
NAFLD and NASH (human data) have increased endotoxin levels [88].
High Fructose diet (Monkey data), with notably high increase in serological LBP-1 [89]. Can be prevented by antibiotics [90].
High fat diet (mice data) increase gut permeability and LPS levels. Can be reversed by antibiotic treatment [91].
High dose (cancerology) MTX (rat [92] and human [93] data).
Vitamin D deficiency (mice data—deficient mice had 50× higher bacterial infiltration in colon tissue) [94].
Acute psychological stress and corticotropin (human [95]).
**Improving Function (Normal Permeability)**
High dose of specific probiotics (mice data) [96].
Anti-TNF-a medication (human data) [97].
Divertin (drug which blocks MLCK1 recruitment); mice data; is more effective than TNFi in colitis models [98].
Omega-3 derived resolvin (mice) [99].
Butyrate (mice data) [68,100].
Cannabinoid CB1 receptor agonist (mice data) [68,101].
Larazotide acetate (Zonulin receptor antagonist) (mice data) [68].
Dietary fibers in RA patients reduced serum zonulin and calprotectin (human data) [102]
